# Genetic diversity of expressed *Plasmodium falciparum var* genes from Tanzanian children with severe malaria

**DOI:** 10.1186/1475-2875-11-230

**Published:** 2012-07-16

**Authors:** Joseph Mugasa, Weihong Qi, Sebastian Rusch, Matthias Rottmann

**Affiliations:** 1 Ifakara Health Institute, Ifakara, Tanzania; 2 Swiss Tropical and Public Health Institute, Socinstrasse 57 Postfach, Basel 4002, Switzerland; 3 University of, Basel, Switzerland; 4 Present address: Genome centre, Zürich, Switzerland

**Keywords:** *Plasmodium falciparum*, Severe malaria, *var genes*, PfEMP1, Expression, Diversity

## Abstract

**Background:**

Severe malaria has been attributed to the expression of a restricted subset of the *var* multi-gene family, which encodes for *Plasmodium falciparum* erythrocyte membrane protein 1 (PfEMP1). PfEMP1 mediates cytoadherence and sequestration of infected erythrocytes into the post-capillary venules of vital organs such as the brain, lung or placenta. *var* genes are highly diverse and can be classified in three major groups (ups A, B and C) and two intermediate groups (B/A and B/C) based on the genomic location, gene orientation and upstream sequences. The genetic diversity of expressed *var* genes in relation to severity of disease in Tanzanian children was analysed.

**Methods:**

Children with defined severe (SM) and asymptomatic malaria (AM) were recruited. Full-length *var mRNA* was isolated and reversed transcribed into *var cDNA.* Subsequently, the DBL and N-terminal domains, and up-stream sequences were PCR amplified, cloned and sequenced. Sequences derived from SM and AM isolates were compared and analysed.

**Results:**

The analysis confirmed that the *var* family is highly diverse in natural *Plasmodium falciparum* populations. Sequence diversity of amplified *var* DBL-1α and upstream regions showed minimal overlap among isolates, implying that the *var* gene repertoire is vast and most probably indefinite in endemic areas. *var* DBL-1α sequences from AM isolates were more diverse with more singletons found (p<0.05) than those from SM infections. Furthermore, few *var* DBL-1α sequences from SM patients were rare and restricted suggesting that certain PfEMP1 variants might induce severe disease.

**Conclusions:**

The genetic sequence diversity of *var* genes of *P. falciparum* isolates from Tanzanian children is large and its relationship to disease severity has been studied. Observed differences suggest that different *var* genes might have fundamentally different roles in the host-parasite interaction. Further research is required to examine clear disease-associations of *var* gene subsets in different geographical settings. The importance of very strict clinical definitions and appropriate large control groups needs to be emphasized for future studies on disease associations of PfEMP1*.*

## Background

Despite all efforts to curb *Plasmodium falciparum* malaria infections it still is an important cause of morbidity and mortality in many developing countries, with an estimated 700,000 deaths annually [1]. The burden of disease is highest in children below five years of age where much of the mortality is attributable to severe malaria. Except for RTS,S, which is currently in clinical phase 3 trial, no vaccine is available to date, but vaccine development is encouraged by the fact that children living in endemic areas attain conditional immunity to severe malaria after a relatively few number of episodes during childhood [2]. Genetic diversity of different parasites and antigenic variation of surface antigens pose an obstacle for vaccine development.

Severe malaria, the most life threatening form of the disease is believed to be mediated by cytoadhesion of *P. falciparum*-infected erythrocytes to a variety of receptors on the endothelial lining of the host’s blood capillaries. This post-capillary sequestration severely affects vital organs such as the brain, kidneys, lungs or placenta [3]. Cytoadherence is conferred by *P. falciparum* derived proteins on the surface of infected erythrocytes that play a key role as both virulence factors and as targets of naturally acquired immunity [4,5]. Of these, the major contributor to pathology of *P. falciparum* is the *P. falciparum* erythrocyte membrane protein 1 (PfEMP1). PfEMP1 is a large protein of approx. 200–400 kDa, it is a highly polymorphic antigen which is encoded by a family of ~60 *var* genes per haploid genome [6]. *Var* genes present with a two-exon structure encoding a semi-conserved C-terminus that contains a predicted transmembrane region, and a highly polymorphic extracellular N-terminus. This part has a modular structure containing various numbers of Duffy-binding-like (DBL) domains and cysteine-rich domains that have been shown to be involved in sequestration of the infected erthrocytes [7-9]. The most N-terminal sequence including the DBLα domain is the most conserved domain within the *var* gene domains also conferring cytoadherence [10,11]. A range of host receptors have been shown to interact with PfEMP1 thus determining the binding to various tissues [10,12,13]. Rosetting and sequestration conferred by expression of different PfEMP1 molecules has been implicated in severe disease [14].

*var* genes have been classified into three major groups (A, B and C) and two intermediate groups (B/A and B/C) based on the presence of one of the 5′ upstream sequences (upsA, B or C), and the position and orientation of the gene within a genomic context [15,16]. It has been speculated that severe malaria is determined by the expression of a restricted and antigenically semi-conserved subset of PfEMP1 [17,18]. The best understood host-parasite interaction concerning PfEMP1 is pregnancy-associated malaria (PAM) in which PfEMP1 molecules binding to CSA are involved [19]. *var* genes have further been sub-classified by the distribution of cysteines throughout the head structures and positions of limited variation (PoLV) [20].

Few studies have investigated the expression of *var* genes in field isolates representing different forms of severe malaria [21-27]. These studies suggested that the transcription patterns of *var* genes vary between different malaria manifestations. Differences in epidemiology, severe disease classification, and *var* classification have also made comparison between studies difficult. Using quantitative real time reverse transcription PCR (qRT-PCR), we have previously shown that group A and B *var* transcripts were up-regulated in children from Tanzania with severe malaria as opposed to asymptomatic infections [25]. Although qRT-PCR is a standard method for detection and quantification of gene expression levels [28], without subsequent sequencing this technique is not informative to study diversity of genes. Given the importance of immunity against PfEMP1 and its possible association with protection against malaria, it is essential to gain amore detailed understanding of diversity of these molecules at sequence level. Only this allows the determination of how such diversity influences the development of protective immunity. In this study, the genetic diversity of expressed PfEMP1 molecules in parasite populations directly isolated from children with severe malaria was examined.

## Methods

### Sample collection

Samples used in this study were collected in a severe malaria (SM) case control study that has been described in detail previously [25]. Briefly, children aged <59 months admitted with severe malaria according to WHO guidelines [29] at Saint Francis Referral Hospital (StFRH), Ifakara, Tanzania were recruited into the study after informed consent was obtained from children’s parents or guardians. Exclusion criteria were: confirmed co-infections, malnutrition (mid-upper arm circumference [MUAC] of ≤12 cm), haemoglobin ≤5 g/dL, lactate ≥5 mmol/L, glucose ≤2.2 mmol/L, or anti-malarial treatment during the last 14 days. A total of 52 patients with SM were recruited. From eight patients meeting the inclusion criteria, cDNA could be obtained and these were grouped as cerebral malaria cases according to WHO guidelines [29] and the modified blantyre coma score ≤3 [30]. Controls were children from nearby villages with asymptomatic malaria (AM) defined as presence of *P. falciparum*, axillary temperature of ≤37.5°C and no other symptoms. Children aged <59 months were screened for *P. falciparum* infection by using a rapid diagnostic test (RDT), (Paracheck® Pf, Orchid Biomedical Systems, Goa, India). Participating children who were found positive by RDT were subsequently confirmed microscopically by Giemsa-stained thick and thin blood film at IHI laboratory. A total of 19 children were initially recruited into the control group, of which only seven could be confirmed *P. falciparum* positive by microscopy. Ethical clearance for this study was obtained from the Ifakara Health Institute and the Medical Research coordinating committee of the National Institute for Medical Research in Tanzania.

From all participating children, one to two ml of venous blood was drawn into an EDTA tube (Vacutainer, Becton Dickinson, Rutherford, NJ, USA). Between 50 – 100 μL of whole blood was immediately mixed with 2 volumes of 6 m guanidine HCl, 50mMTris pH 8.0, 20 mM EDTA and kept at −20°C for gDNA isolation. The remaining erythrocytes (RBC) were separated from serum by centrifugation and washed with 40 ml phosphate buffered saline, 5 volumes TRIzol reagent (Invitrogen) were added to the RBC pellet before preservation at −70°C until later use.

### DNA extraction and genotyping

Genomic DNA was extracted from frozen blood in guanidine HCl using QiaAmp blood kit (Qiagen) following the manufacturer’s instructions. The minimum number of genotypes per isolate was determined by m*sp2* PCR amplification and subsequent Genescan analysis as described by Falk *et al.*[31]. Briefly, 1 μL of purified genomic DNA was used in a 20 μL primary PCR reaction, followed by a nested PCR reaction using fluorochrome-labelled primers for both m*sp2* allelic families. Capillary electrophoresis was used to determine the number of infecting strains per isolate.

### Isolation of full-length *var* transcripts and RT- PCR

Total RNA was extracted by using TRIzol reagent (Invitrogen) twice as recommended by the manufacturer to decrease DNA contamination. Between TRIzol purifications RNA was treated with 3 U of RQ1 RNase-free DNase (Promega). Full-length *var* mRNA was isolated by using magnetic beads tagged with an oligonucleotide complementary to the acidic terminal sequence (ATS) as previously described [23] with modifications. Briefly, RNA was dissolved in 5 mM Tris, 0.5 mM EDTA, and mixed with binding buffer (0.5 M LiCl, 1 mM EDTA, 10mM Tris, pH 7.5), 15 mM DTT, 40 U RNaseOUT (Invitrogen) and 1 pmol of biotinylated oligonucleotide complementary to the conserved sequence in the ATS domain (Biotin-5*'*-GGTTC(A/T)A(A/G)TAC(C/T)ACTTC(A/T)AT(C/T)CCTGGT(A/G)CATATATATCATTAATATCCAATTCTTCATA(C/T)TCACTTC(T/G)GA(A/T/G)GA-3′). This mixture was incubated 65°C for 30 minutes and afterwards kept at 4°C. Meanwhile 150 mg of Dyna beads m-280 streptavidin (Dynal Biotech, ASA, Oslo, Norway) was washed as suggested by the manufacturer and resuspended in 0.5 M LiCl and added to the oligonucleotide-RNA hybrids. The mixture was uniformly mixed by rotating for 30 min at 37°C. The biotinylated beads-ATS-mRNA complex was washed three times with washing buffer (10 mM Tris, 1 mM EDTA, 0.15 mM NaCl, pH 7.5) and once with 10 mM Tris. Reverse transcription (RT) into single stranded cDNA was performed on captured mRNA, primed by random hexamere oligonucleotides in the concentration of 300 ng (Invitrogen) using Sensiscript reverse transcriptase (Qiagen) following the manufacturer’s protocol in a final volume of 20 μL. A second RNA aliquot was treated equally but reverse transcriptase was omitted in the RT-step. This sample served as a control for proving the absence of gDNA. After RT, cDNA was treated with RNaseA (Promega) and 1 μL was used for subsequent PCR analyses.

### Amplification of *var* sequences

The DBL-1α domain of *var* genes was PCR amplified from 1 μL cDNA using Advantage cDNA polymerase mix (CLONTECH) and the primer sets shown in Table 1. PCR was performed in 25 μL 1.5 mM MgCl_2_, 200 μM dNTP mix, 1 μM each primer. The cycling conditions were 30 cycles of 94°C for 30 s, 1 min at the annealing temperature specified in (Table 1) and for 70 s at 68°C. This generated a PCR product of about ~ 400–500 bp in length.

**Table 1:** **Oligonucleotide primers used for amplification of different fragments of *var *genes**

***var* Gene region**	**Size of amplified product**	**T_anneal_**	**Name of primer**	**Primer sequence**	**Source**
DBL1α	~500	54	DBLα-5′	5′-GCACGAAGTTTTGCAGATAT(A/T)GG-3′3′-	[23]
			DBLα-3′	AA(A/G)TCTTC(T/G)GCCCATTCCTCGAACCA-5′	
DBL1α-CIDR	1.5 kb	52	DBLα-5′	5′-GCACGAAGTTTTGCAGATAT(A/T)GG-3′	[23]
			CIDR1.1-3′	3′-T(C/G/T)TAGTAATTTATC(A/C/T)ATTGT-5′	
			CIDR1.2-3′	3′-T(C/G/T)TAATAAGAATTCGATTGC-5′	
upsA 5′UTR- DBL1α	1.2 kb	54	upsA-5′	5′-ATTA(C/T)ATTTGTTGTAGGTGA-3′	
			DBLα-3′	3′-AA(A/G)TCTTC(T/G)GCCCATTCCTCGAACCA-5′	
upsB 5′UTR- DBL1α	1.3 kb	52	17DBLα-5′	5′-ATGTAATTGTTGTTTTTTTTTTTGTTAGAATATTTAAA-3′	
			DBLα-3′	3′-AA(A/G)TCTTC(T/G)GCCCATTCCTCGAACCA-5′	
psC 5′UTR- DBL1α	1.3 kb	54	5B1-5′	5′-CACATATARTACGACTAAGAAACA-3′	[15]
			DBLα-3′	3′-AA(A/G)TCTTC(T/G)GCCCATTCCTCGAACCA-5′	[23]

Three different PCRs were carried out on cDNA to determine the genetic diversity in the upstream sequences using three degenerate forward primers based on sequence alignments of 3D7 *var* genes (Table 1). These primers amplify homology blocks in the upstream sequences with following sizes: 200 bp for upsA, 400 bp for upsB, and 440 bp for upsC. The reverse primer was chosen from the homology block H of the first DBL-1α domain [32]. Primers were tested on genomic DNA from the 3D7 isolate. Amplification of DBLα-CIDRβ fragments was carried out using primers shown in Table 1. Controls without reverse transcriptase were amplified in parallel for each reaction, and if a PCR product was obtained the RT(+) sample was discarded and excluded from the analysis.

### Cloning and sequencing

An aliquot of 5 μL of each PCR product was visualized in a 1% agarose gel, the remaining PCR product was purified using the NucleoSpin® Extract II kit (Macherey & Nagel). The eluted DBL-1α fragments were cloned into the pGEM-T vector (Promega) according to the manufacturer’s instructions and transfected into *E.coli* SURE cells (Stratagene). 5′ upstream regions and DBL-1α-CIDR1 products were cloned into the pCR®4-TOPO vector (Invitrogen) and transformed into *E.coli* TOP10 cells. This vector was more suitable for large fragments (> 1 kb) and for PCR products with low concentration (< 5 ng/μL). From each cloning reaction an average of 50 colonies found positive by PCR screening were further processed for sequencing using the Perfectprep® Plasmid 96 Vac Direct bind kit (Eppendorf). The size of each insert was checked from purified plasmids using restriction enzymes NotI and NcoI (New England BioLab) for pGEMT plasmids. EcoRI digests (New England BioLab) were used for TOPO plasmids. Sequencing was carried out using the T7 and SP6 primers for pGEM-T vector, whereas M13 forward and reverse primers were used for the pCR®4-TOPO using a 96 capillary automated sequencing systems 3700 (Applied Biosystems). A multiple-sequence alignment of sequences derived from the same clinical isolate was carried out to allow the exclusion of PCR derived mutations. Two sequences were considered to be identical when ≥96% amino acid sequence identity was detected.

### Sequence analysis

DNA sequences were assembled and analyzed using ContigExpress in the Vector NTI Advance™ 10 software (Invitrogen) and BLAST from the NCBI webpage (http://www.ncbi.nlm.nih.gov/BLAST/). BLAST analysis against the 3D7 genome database was performed using the PlasmoDB interface (https://www.pasmoDB.org[33]). DNA sequences were translated using RevTrans1.4 [34]. Amino acids were aligned with either CLUSTALW 1.8 or MUSCLE [35] using default parameters and edited with Bioedit version 7.09 with minor manual adjustment where necessary. Sequences were further categorized into sequence types (STs) by BLASTCUT analysis [36] by which sequences sharing ≥96% sequence identity were assigned the same ST.

### Phylogenetic analysis

Phylogenetic analyses were conducted on multiple sequence alignments of the 3 most dominant sequences from each isolates. Because *var* genes are subject to intragenic recombination [37], synonymous substitutions are likely to be saturated and DNA sequence analysis would be quite noisy in constructing phylogenetic trees [38]. Therefore, we used protein sequences rather than nucleotide sequences. Two methods were employed in constructing the phylogenic trees. Neighbour-Joining (NJ) trees were constructed by using MEGA 4.0 [39]. The reliability of internal branches for NJ was assessed with 1,000 bootstrap pseudo-replicates using ‘pairwise deletion option’ of amino acid sequences with p-distance. SplitsTree4 version 4.7 was used to construct the phylogenetic network [40] using the Neighbour-Net distances transformation and equal angle splits transformation.

## Results

### Sample collection and clinical data

A total of 15 children were used in the analysis of the present study of which 8 were in the SM group with cerebral manifestation, Blantyre score ≤3 and 7 children with asymptomatic *P. falciparum* malaria were in the control group. Clinical and epidemiological assessments of all subjects are presented in Table 2. There was a significant difference in age between AM cases (median 52, range 24–59) and SM cases (median 28.5, range 14–40, *p = 0.02*, Kruskal-Wallis test). There was also a highly significant differences in parasitaemia between both clinical categories (*p* =0.0012, Kruskal-Wallis test).

**Table 2:** C**linical and epidemiological assessment of isolates from severe and asymptomatic malaria**

**Isolate**	**Sex**	**Age (months)**	**Days between symptoms and treatment**	**Parasitemia (parasites/200 leukocytes)**	**Temperature (°C)**	**MUAC (cm)**	**PCV%**	**Lactate mmol/L**	**Glucose mmol/L**	**Blantyre score**
Severe										
ISM2	M	40	3	3120	38.6	17	27	2.5	2.3	2
ISM3	M	36	2	2574	39.7	17	16	2.4	5.9	3
ISM11	M	33	2	7344	37.0	17	30	4.5	3.7	3
ISM16	M	16	3	2484	38.6	14	26	2.1	2.7	3
IMS33	F	24	3	1316	37.4	16	22	3.1	3.0	2
ISM48	M	36	2	4713	38.9	17	31	3.0	5.0	3
ISM49	M	14	4	1907	39.9	16	23	1.8	6.6	3
ISM51	F	16	4	9999	40.0	16	21	5.0	8.4	2
Asymptomatic										
IAM5	M	24	NA	830	36.7	ND	20	3.4	5.8	NA
IAM7	F	33	NA	704	36.6	ND	14	3.3	3.9	NA
IAM10	M	59	NA	70	37.5	ND	22	2.4	4.2	NA
IAM11	F	56	NA	68	36.7	ND	23	4.6	4.4	NA
IAM12	F	47	NA	690	36.4	ND	26	4.5	8.0	NA
IAM17	M	59	NA	360	37.3	ND	28	2.5	4.9	NA
IAM18	F	52	NA	360	37.3	ND	18	3.1	4.3	NA

NA Not applicable, ND Not determined.

### Multiplicity of infection

*msp2* genotyping indicated that 87.5% (7/8 SM isolates) had multiple *P. falciparum* clone infections (2–4clones) with an average of 2.6 infecting clones. All isolates from AM children had multiple infections (2 or 3) with an average of 2.4 infecting clones (Table 3).

**Table 3:** **Summary of analysed sequences of different transcribed *var *DBL1α sequences**

**Isolates**	**RT-PCR, cloned in pGEMT Vector, PCR screened 96 clones picked for sequencing**	**Sequences generated per isolate**	**Number of distinct DBLα *var* per isolate**	**Predominant gene blasted vs 3D7**	**Group homology to 3D7**	**Bulls’ signature and group**	**MOI**
Severe							
ISM 2	48	42	23	PF08_0141	A	LFLG-IREY-KAIT-2-LTNL	2
ISM 3	48	41	22	PFF0010w	B/A	LYLD-FREY-KAIT-2-PTNL	3
ISM 11	60	50	20	PFD1005c	B/C	LFIG-LRED-KALT-4-PTYF	2
ISM 16	48	36	18	PFD0020c	A	MFKR-LRED-RAIT-2-PTNL	1
ISM 33	60	50	15	PFF0010w	A	LFLG-VREY-KAIT-2-LTNL	3
ISM 48	48	47	12	PF08_0141	A	MFLG-IREY-KALT-2-PTNL	3
ISM 49	48	44	17	PFL1830c	B	LYLG-LRED-KAIT-4-PTYF	3
Asymptomatic							
IAM 5	48	46	25	PF08_0141	A	MFLG-IREY-KALT-2-PTNL	3
IAM 7	48	45	8	PFL 1830c	B	LYLG-LRED-KALT-4-PTYF	2
IAM 10	48	38	23	PFD0615c	C	LFIG-LRED-EAIT-4-PTNF	3
IAM 11	48	43	15	PFL2665c	B	LYRG-LRED-NAII-3-LTNF	3
IAM 12	48	45	20	PFL1955w	B/C	LYLG-LRED-KAIT-4-PTYF	2
IAM 17	48	46	24	PFA0005w	B	LYLG-LRED-EAIT-4-PTYF	2
IAM 18	48	42	16	PFA0005w	B	LYLG-LRED-KAIT-4-PTYF	2
	Total	615					

### DBL-1α sequence types

A total of 615 *var* DBL-1α clones (~ 400–500 bp) were successfully sequenced. Of these, 305 sequences were originating from AM children (Table 3) and were assembled into 131 sequence types (STs) *i.e.* distinct DBL-1α sequences. The remaining 310 sequences from SM children were assembled into 127 sequence types. AM patient samples had more singletons (sequences occurring only once) than SM isolates (p<0.05).

Assembled sequence types showed an extreme diversity in sequence reflecting the high recombination and mutation rates in the DBL-1α domain. Multiple-sequence alignment of the DBL-1α sequences showed conserved islands of homology. The dominant sequence from each isolate was blasted against the 3D7 genome. The blasted sequence was assigned the name of the identified 3D7 gene with the high scoring segment pair (Table 3). PCR amplification and cloning efficiency of the *var* up-stream regions (upsA, upsB, upsC and DBL1α-CIDR) were very low and few sequences could be generated. These sequences were, therefore, excluded from the analysis.

### Distribution of DBL-1α expressed sequence tags

**Figure 1:**
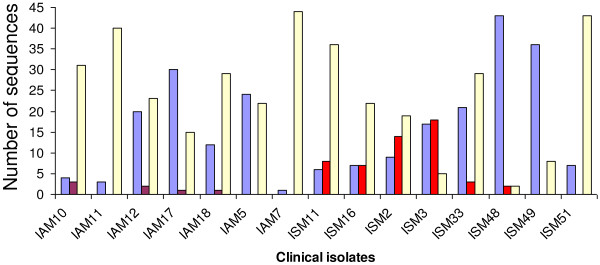
**Distribution of unique sequence types (STs) of DBL1α in clinical isolates. **Blue columns represent STs found in multiple samples from both AM and SM groups. Red columns represent STs found in multiple samples within the SM group. Pink columns represent STs found in multiple samples within the AM group. Yellow columns represent STs specific to each individual isolate.

The number of distinct transcribed DBL-1α *var* gene sequences detected per isolate varied from 8 to 25 (Table 3). All isolates showed a predominant sequence as well as minor transcripts and unique sequence types. The homologous group A transcript to PFD0020c in 3D7 was a predominant transcript in ISM 16 and was among the top three frequent sequences in SM isolates. However, the transcript homologous to PF08_0141 in 3D7 was the predominant transcript in 2/7 SM isolates (Table 3). In cluster analyses some of the DBL-1α sequences were found to be shared among isolates (*i.e* overlapping). A number of transcripts were found in both groups (SM & AM) and others were specifically found either in SM or in AM isolates. Some sequences were unique to a particular isolate and were not found in other isolates. The distributions of the STs in our 15 isolates are shown in Figure 1. There was no significant difference in the number of distinct DBL-1α sequences per isolate detected in both clinical groups AM: median 20, range 8–25, SM: median 17.5, range 12–23, (*P* = 0.72, Kruskall-Wallis test).

### Distribution of DBL-1α expressed sequences tags in clinical isolates

**Figure 2:**
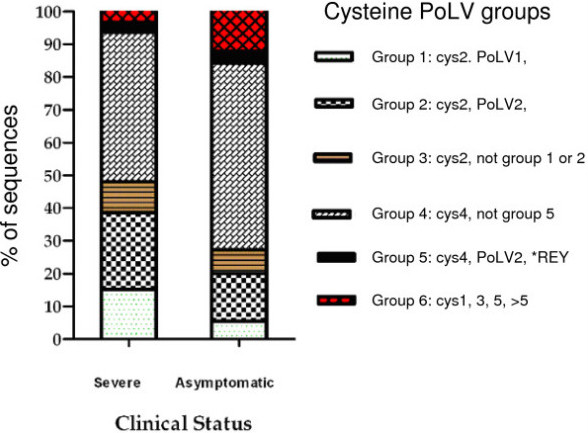
**Distribution of DBL-1α sequences into cys/PoLV groups by clinical status. **SM DBL-1α sequences had more than 50% cys2 sequence tags (1–3 groups) compared to 27% in AM isolates.

**Figure 3:**
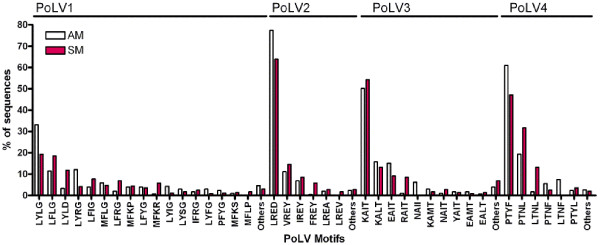
Distribution of PoLVmotifs within clinical isolates SM (red bars) and in AM (white bars).

All sequences generated were classified into six DBL-1α sequence tag groups by using text string software in MS Excel and Perl which was kindly provided by Dr P Bull (KEMRI, Kilifi, Kenya). This classification of DBL-1α sequence tags was previously explained in detail by Bull *et al.*[20]. In summary, it is based on counting the number of cysteine-residues within the tagged region, and in a set of sequence motifs at four positions of limited variability (PoLV 1–4). Figure 2 shows the distribution of PoLV/cys groups in clinical isolates. Analysis of DBLα sequences generated in the present study corresponded well with the cysteine/PoLV grouping. Figure 3 shows the proportional distribution of PoLV motifs between the clinical isolates. Sequence ‘signature tags’ and the dominant sequence group from each isolate are shown in Table 3. A significant association of cys2 sequence tags (groups 1–3) with SM isolates (p<0.0001, CMH test), with an odds ratio of 2.5 (95% CI = 1.78-3.4) was observed. These findings support previous reports that DBL-1α sequences associated with severe disease have a reduced number of cysteines [21-23]. A two-sample test of proportion showed that expressed PoLV motifs were associated with a particular disease phenotype (p<0.0001). These PoLV motifs were found strongly associated with severe malaria: in PoLV1 (LDLY and MFKR), in PoLV2 (FREY and LREV), in PoLV3 (NAIT and RAIT), and in PoLV4 (LTNL and PTNL).

### Cumulative diversity of DBL-1α sequences in clinical isolates

**Figure 4:**
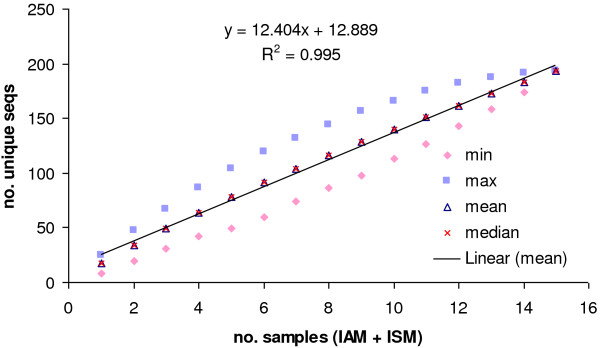
**Cumulative diversity curves for DBL-1α sequences from Ifakara. **The cumulative curve for DBL-1α was determined by simulation the number of unique sequences as a function of the number of patient samples. For each number of patient samples the statistics value was obtained from simulations of all possible sample combinations.

To estimate the size of the *var *gene repertoire in the parasite population under study, the rate at which distinct DBL-1α sequences changed was simulated with increasing sample size. This simulation was performed separately for AM and SM, as well as the combined data (AM & SM). The empirical plots were fitted by a linear function. The curves did not plateau with the DBL-1α sequences generated from the Ifakara area (Figure 4). The repertoire of expressed *var* genes was unlimited. Thus, *var* gene diversity in this local population seems to be immense and unrestricted. However, a minimal overlap among *var* genes was found in different isolates. A similar finding has been reported by Barry *et al.*[41] for the cumulative DBL-1α sequences from genomic DNA in the Amele, PNG, and for the global population, where in more than 1,000 sequences from 59 isolates plus the entire 3D7 *var* repertoire the saturation point of the *var* gene repertoires could not be reached.

### Phylogenetic analysis

**Figure 5:**
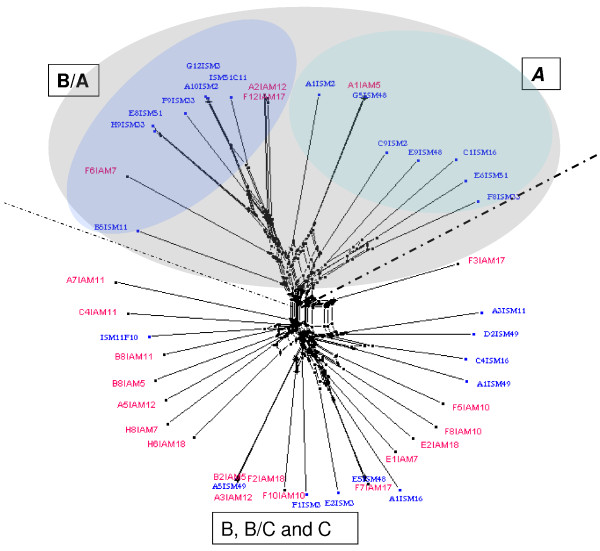
**Phylogenetic network showing the comparison of 3 dominant DBL-1α sequence tags transcribed from clinical isolates, generated using Neighbour-Net **[44]**. ** Sequences transcribed by isolates with severe malaria (ISM, blue) and asymptomatic malaria (IAM, red) are compared. The sequences fall into two major clades separated with dotted lines, the upper cluster formed 2 subgroups of sequences isolated from severe patients, one with group A and the remaining *var* group B/A homology to 3D7. The majority of sequences from asymptomatic patient isolates clustered together and were homologous to group B, B/C and C of the 3D7 genome.

**Figure 6:**
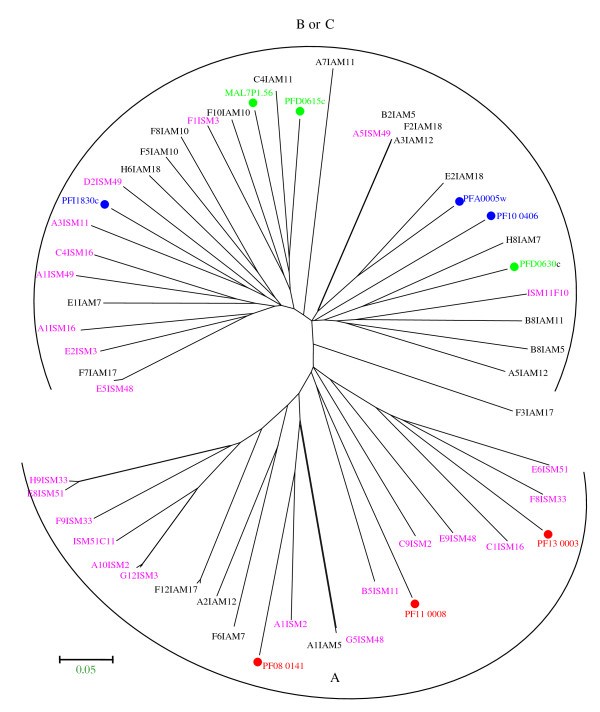
**Phylogenetic comparison of 3 dominant DBLα sequence tags transcribed from each clinical isolates and selected tags from 3D7.** A neighbor-joining tree was generated using amplified DBL-1α fragments. The bootstrap consensus tree inferred from 1000 replicates [45], using pairwise deletion of amino acid sequences with p-distance. Phylogenetic analyses were conducted in MEGA4 [39]. Sequences transcribed by isolates from children with severe malaria (ISM, pink), asymptomatic malaria (IAM,black) and 3D7 genes (group A, red; group B, blue; group C, green).

To study the sequence diversity between the SM and AM groups, a phylogenetic network was constructed using the three most dominant DBL-1α variants expressed from each SM or AM isolate. The analysed sequences clustered in two distinct groups. The majority of the DBL-1α isolates from severe malaria clustered together and belonged to *var* group A and B/A. AM isolates formed another cluster mainly consisting of *var* B, B/C or C (Figure 5). To study further relationships between sequences, nine DBL-1α sequences from the 3D7 genome were incorporated, three from each group A, B and C, in the construction of the phylogenetic tree. Among the sequences included was the group A *var* PF11_0008 which has been shown to be highly transcribed in the NF54 isolate [42] and group B *var* PF10_0406 which has been detected previously as a major transcript in 3D7B2 and 3D7B1 samples [43]. The DBL-1α sequences analysed were found to cluster into two distinct clades. The majority of SM isolates and the 3D7 DBL-1α sequences clustering together belonged to *var* group A. AM isolates and the other remaining 3D7 DBL-1α sequences formed another cluster mainly belonging to *var* group B, B/C or C (Figure 6).

These findings again support the hypothesis that SM is caused by a restricted subset of *var* genes that belong to *var* group A or B/A whilst non severe malaria is attributed to the presence of another *var* gene group. Similarly, the phylogenetic approach of Kyriacou *et al.*[21] using DBL-1α sequence tags from Mali identified *var* transcripts from group A and B/A genes to be more frequent among parasites isolated from children with cerebral malaria than from patients with hyper-parasitaemia.

**Figure 7:**
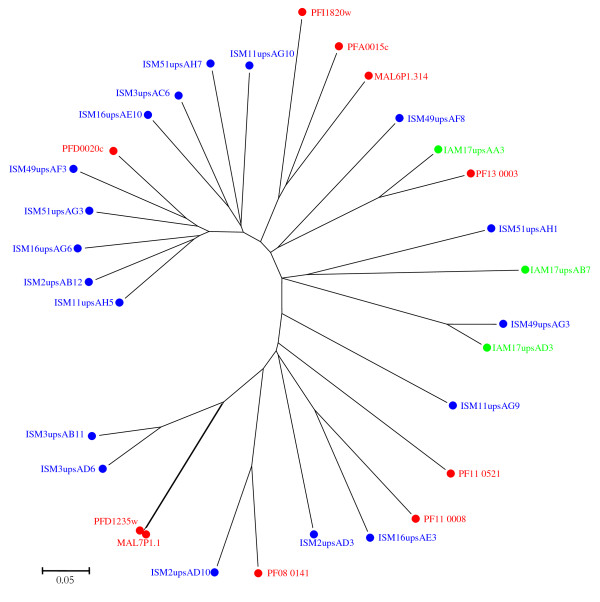
**Phylogenetic comparison of *var *group A from 3D7 genome and 3 dominant *var *group A amplified from clinical isolates. **A neighbor-joining tree was generated based upon the predicted protein start site in the N-terminal segment (NTS) domain to the first DBL-1α H block. The bootstrap consensus tree inferred from 1000 replicates [44], using pairwise deletion of amino acid sequences with p-distance. Phylogenetic analyses were conducted in MEGA4 [39]. Sequences transcribed by isolates from children with severe malaria (ISM, blue), asymptomatic (AS, green).

A multiple-sequence alignment of three dominant upsA sequences from clinical isolates together with upsA sequences from the 3D7 genome showed the existence of short islands of homology, conserved in all isolates suggesting that they might be structurally important. Phylogenetic analysis of the three dominant upsA sequences from clinical isolates and the 3D7 upsA sequences, showed an even distribution among clinical isolates (Figure 7). Two different methods for phylogenetic tree construction were used (MEGA 4.0 and SplitsTree 4.7) and both methods yielded similar tree topologies.

## Discussion

Studies on *var* gene diversity are important in understanding malaria pathogenesis and in the design of disease interventions such as a vaccine or chemotherapies. In the present study, we examined *var* gene expression from clinical isolates of children with severe malaria and asymptomatic infections from Tanzania. In each isolate dominant expression of one particular *var* gene was found, together with less abundant variant transcripts and unique sequences. However, the dominant sequences differed between isolates. This suggests that each parasite contains its own set of *var* gene variants. This has the consequences that exposure to multiple infections and hence *var* gene products do not necessarily confer immunity to future malaria infections [46,47].

By analysing the expressed *var* gene repertoires in severe malaria cases versus asymptomatic controls, we showed that the diversity within the *var* gene family is enormous with a minimal degree of overlaps between isolates. Kyriacou *et al.*[21] have found a minimal overlap in *var* gene repertoires after analysing the expressed sequence tags from Malian children with malaria infections. A recent study on molecular epidemiology of *var* genes in Africa has also shown a minimal overlap in *var* repertoires among parasite genomes [48]. In contrast, Albrecht *et al.*[49] reported a large overlap of the *var* gene repertoire in Western Amazon isolates. *var* repertoires of natural parasite populations found within specific geographical regions showed a degree of overlapping, suggesting the circulation of a similar *var* gene repertoire. This has important implications for the acquisition of long-term immunity by the exposed individuals [47].

The diversity of *var* genes within a natural *P*. *falciparum* population in a particular geographical region is difficult to define, and to assess whether the diversity is constant due to functional constrain on this molecule, fluctuating or constantly turning over, and how fast the turnover rate of the PfEMP1 repertoires could be. Changes in the *var* repertoire are believed to be due to high allelic and ectopic recombination rates of *var* genes in field isolates [37,50,51] which are influenced by transmission intensity. The diversity of the PfEMP1 repertoire of parasites in a given geographical area is a key factor in the development of clinical immunity. The vast antigenic diversity and complexity of *var* gene repertoires in parasite populations may explain why individuals are repeatedly susceptible to *P. falciparum* infections and never develop sterilizing immunity. The antigenic variation and high switching rate of *var* gene expression are effective mechanisms adopted by *P. falciparum* to evade the host’s immune system, for survival, and effective transmissions.

In this study, several sequences were observed more frequently than others within individual patients. This is consistent with previous studies of *var* gene diversity [21,22,46,50,51]. The variability of the DBL-1α and upstream sequences within an isolate was found to be similar to different isolates in both the groups (SM & AM). AM isolates were more diverse as reflected by the presence of more singletons suggesting that *var* genes associated with asymptomatic infection have an enormous repertoire which could explain the difficulty of acquiring immunity to mild or asymptomatic malaria.

Isolates from children with severe malaria were predominantly found to transcribe *var* genes with a DBL-1α domain that had a reduced number of cysteine residues which is the characteristic of *var* group A. Similar results have been reported previously from other research groups in Kenya, Mali, and Brazil [20-22]. This supports the notion that severe malaria might be caused by a restricted subset of *var* genes and confirms that group A *var* genes are involved in severe disease similarly as we had shown in a previous study that group A *var* genes were up regulated in children with cerebral malaria [25]. However, most studies on *var* gene diversity have been relying on the use of DBL-1α fragments [50]. DBL-1α primers amplify only a small fragment of the *var* gene that is more conserved than other *var* domains and that is found in most of PfEMP1 proteins. Due to the complex nature of *var* genes, only recently complete *var* genes could be cloned routinely [52] and could provide in future additional information on understanding *var* gene transcription and its association to disease phenotype.

Cluster analysis revealed several ‘unique sequences’ of *var* genes which were transcribed only in isolates from patients with severe malaria. Expression of these ‘unique sequences’ in a patient who lacks a pre-existing antibody response against this variant might trigger the development of severe malaria. Once exposed to these potentially virulent *var* genes individuals living in endemic areas may acquire immunity to severe malaria. In areas of high endemicity this might happen early in life after only a few clinical episodes. The distribution of PoLV motifs showed 8 motifs which were highly associated with severe disease. Based on the MOTIFF algorithm, Normark *et al.*[53] identified 15 DBL-1α degenerate sequence motifs pertinent to severe disease and three motifs associated with the high rosetting phenotype after analysing 93 patients with well-characterized disease. Once again pointing in the direction that disease phenotypes are correlated with the expression of certain PfEMP1 variants and motifs. This is highly relevant information for vaccine development and understanding disease pathogenesis.

The distribution of PF11_0008, a group A *var* gene, which previously has been identified in the 3D7 genome and the isogenic isolate NF54 [42], was found in three SM isolates (ISM11, ISM33, ISM48) and in one AM sample (IAM17), although in low frequencies. This, and the observation that PFD0020c also has been more frequently found in SM cases suggests that the *var* genes of laboratory strains are shared among the field isolates. The recent report by Claessens *et al.*[54] showing that up-regulation of the group A *var* gene 3D7_PFD0020c is associated with adhesion to human brain endothelial cells further supports this notion.

## Conclusion

The *var* family is highly diverse in natural *P. falciparum* populations, but this diversity was more restricted in severe malaria than in asymptomatic isolates, and this finding suggests a fundamental role played by different subsets of *var* transcripts in disease syndromes. Further analysis of this molecule is required from many geographical regions with well-defined malaria infections. Such an approach might provide the basis for an innovative vaccine or chemotherapy intervention. To gain better understanding of *var* gene diversity and function future work should be focused on analysis of full-length sequences and the analysis of protein function and immunological responses.

## Competing interests

The authors declare no competing interests.

## Authors’ contributions

JM, SR, MR, HPB conceived and designed the study, JM and SR conducted the laboratory analysis, WQ and JM analysed the data. JM and HPB wrote the manuscript with contributions from all other authors. All authors read and approved the final manuscript.
